# Macromolecular Model of the Pectic Polysaccharides Isolated from the Bark of Norway Spruce (*Picea abies*)

**DOI:** 10.3390/polym13071106

**Published:** 2021-03-31

**Authors:** Myriam Le Normand, Barbara Rietzler, Francisco Vilaplana, Monica Ek

**Affiliations:** 1 Division of Wood Chemistry and Pulp Technology, Department of Fibre and Polymer Technology, KTH Royal Institute of Technology, Teknikringen 56, SE-100 44 Stockholm, Sweden; myriamln@kth.se (M.L.N.); rietzler@kth.se (B.R.); monicaek@kth.se (M.E.); 2 Wallenberg Wood Science Centre (WWSC), KTH Royal Institute of Technology, Teknikringen 56, SE-100 44 Stockholm, Sweden; 3 Division of Glycoscience, Department of Chemistry, KTH Royal Institute of Technology, AlbaNova University Centre, SE-106 91 Stockholm, Sweden

**Keywords:** pectic polysaccharides, spruce bark, pressurized hot-water extraction, biorefinery, size-exclusion chromatography

## Abstract

The bark of Norway spruce (*Picea abies*) contains up to 13% pectins that can be extracted by pressurized hot water, which constitute a valuable renewable resource in second-generation lignocellulosic biorefineries. This article proposes, for the first time, structural molecular models for the pectins present in spruce bark. Pectin fractions of tailored molar masses were obtained by fractionation of the pressurized hot water extract of the inner bark using preparative size-exclusion chromatography. The monosaccharide composition, average molar mass distribution, and the glycosidic linkage patterns were analyzed for each fraction. The pectin fraction with high molecular weight (*M_w_* of 59,000 Da) contained a highly branched RG-I domain, which accounted for 80% of the fraction and was mainly substituted with arabinan and arabinogalactan (type I and II) side chains. On the other hand, the fractions with lower molar masses (*M_w_* = 15,000 and 9000 Da) were enriched with linear homogalacturonan domains, and also branched arabinan populations. The integration of the analytical information from the macromolecular size distributions, domain composition, and branch lengths of each pectin fraction, results in a comprehensive understanding of the macromolecular architecture of the pectins extracted from the bark of Norway spruce. This paves the way for the valorization of spruce bark pectic polymers in targeted applications based on their distinct polymeric structures and properties.

## 1. Introduction

The bark of Norway spruce (*Picea abies*) is one of the largest by-products of the Scandinavian forest industries, representing around 1–1.5 million tons/year in Sweden or Finland alone [1,2]. This abundant biomass is commonly used as a fuel to produce heat and electrical energy in the mill. Recently, there has been growing interest in converting industrial bark into added-value products as part of the biorefinery approach. This strategy requires a good understanding of the chemical composition of the bark and a deep knowledge of the macromolecular structure of its individual components. The bark contains considerable amounts of bioactive components such as antioxidants (polyphenols), and also structural polysaccharides such as pectins. Efficient pectin extraction and recovery would contribute to the integral valorization of this biomass resource through the design of cascade biorefinery processes resulting in a multiple product portfolio.

The pectin content in spruce bark has been estimated to be 9.0% in the outer bark, 12.6% in the inner bark, and 10.5% in the bark collected in a paper mill directly after the debarking process. [3,4,5]. Pectins have also been identified in the bark of various conifer species such as black spruce [6], white spruce [7], Pacific silver fir [8], and in the bark of Scots pine [9]. Nevertheless, most of these studies have been limited to a simple report of the carbohydrate composition of the bark and no real attempt dealing with the structural characterization of bark pectin at the different macromolecular hierarchical levels has been undertaken.

Pectins are heterogeneous polysaccharides that are present in the primary cell walls and middle lamellae of all plants [10]. Pectins are composed of at least 17 different monosaccharides, with d-galacturonic acid (GalA) as the most abundant followed by d-galactose (Gal), l-arabinose (Ara) and l-rhamnose (Rha) [11,12]. However, their structure is believed to follow a general model based on four structurally distinct domains, including homogalacturonan (HG), a linear polymer of α-1,4 linked GalA, rhamnogalacturonan I (RG-I) and rhamnogalacturonan II (RG-II) and some xylogalacturonan (XGA) chains [13]. RG-I is composed of a polymeric backbone of alternating Rha and GalA units with neutral side chains of Ara and Gal (arabinans and arabinogalactans). The RG-II domain consists of a well-conserved HG backbone branched with complex side chains containing eleven different monosaccharides, including some minor sugars as 2-O-methylxylose, 2-O-methylfucose, apiose, aceric acid, 2-keto-3-deoxy-d-manno-octulosonic acid, and 3-deoxy-d-lyxo-2-heptulosaric acid [14,15]. These domains are considered to be covalently linked and form a complex pectic network [16,17]. The branched regions (RG-I, RG-II, XGA) are referred to as the “hairy regions” and the linear HG as the “smooth region”. The properties of pectin make it a popular material to be used in the food industry as emulsifier, thickener, gelling agent, and stabilizer [18,19,20]. Pectins also show potential immunostimulant properties and have possible applications in wound dressing, tissue engineering and drug delivery [21,22,23].

Pressurized hot water extraction, i.e., extraction using water in liquid state at high temperature and pressure, emerges as a sustainable technology for the recovery of polysaccharides from recalcitrant lignocellulosic tissues [24,25,26,27]. It allows for a mild extraction without extensive depolymerization of the biopolymers, so it can be further utilized in films or hydrogels. There is also potential to scale up this kind of extraction for industrial use [24,25,28]. Pressurized hot water at 100–140 °C enables the extraction of significant amounts of pectins from the inner bark of Norway spruce [3,5], which exhibit highly branched RG-I domains [4]. This structural characteristic has been confirmed by glycome profiling of cell wall extracts isolated from spruce bark and bark hot water extracts [29]. However, these studies were based on crude extracts or mixture of non-cellulosic polysaccharides and did not allow a precise structural characterization of bark pectins. The aim of this work was to elucidate the complex structure of pectins in the bark of *Picea abies*, providing detailed information of the linkage patterns and the macromolecular architecture. The integration of this information offers a macromolecular model for the pectins from spruce bark.

## 2. Experimental

### 2.1. Material

Bark of Norway spruce was sampled from a fresh 30-year-old tree cut in Gävleborg County (Sweden) in July 2009. It was stored in the dark at −20 °C. Inner and outer bark was manually separated using a scalpel. The inner bark was ground with a hand blender to a particle size of approximately 5 × 2 mm^2^ and smaller without a previous drying step.

### 2.2. Isolation of Pectins from the Inner Bark

The ground inner bark was sequentially extracted in an Accelerated Solvent Extractor (ASE) (Dionex, Sunnyvale, CA, USA) as described by Le Normand et al. [3]. 10 g wet inner bark (dry content of 56%) was placed in 34 mL stainless steel extraction cells, extracted with acetone at 100 °C followed by pressurized hot water extraction in 3 cycles of 20 min at 100 °C followed by 3 cycles of 20 min at 140 °C at a pressure of 100 bar. The fraction obtained after extraction at 140 °C was filtered, dialyzed against distilled water in a Spectra/Por dialysis tube with a molecular mass cut off of 3.5 kDa. Starch was removed from the filtered and dialyzed extracts by treatments with α-amylase from porcine pancreas (Type VI-B, 23 units/mg, Sigma-Aldrich, St. Louis, MO, USA). For this, 50 mg of the freeze-dried extract were dissolved in 30 mL of 0.01 M phosphate buffer (pH 7), stirred in a water bath at 37 °C, and the enzyme (7 mg) was added every 24 h during three days. The solution was then filtered, dialyzed and freeze-dried (Christ ALPHA 2–4 LD plus, Martin Christ GmbH, Niedersachsen, Germany).

### 2.3. Fractionation of the Pectins

The size fractionation of the treated pectin extract was performed on a Dionex Ultimate-3000 liquid chromatography system (Dionex, Sunnyvale, CA, USA), equipped with a 2 mL injection loop, an LPG-4300SD gradient pump and a Waters-410 refractive index (RI) detector (Waters, Milford, MA, USA). Size separation was achieved using a preparative size-exclusion chromatography (SEC) column PSS SUPREMA (300 × 20 mm, porosity 1000 Å, particle size 10 µm) preceded with a guard column PSS SUPREMA (50 × 20 mm^2^, particle size 10 µm). An aqueous pectin solution at a concentration of 13.4 g L^−1^ was injected and eluted with 10 mM NaOH as mobile phase with a flow rate of 1 mL min^−1^ at 30 °C. A total of six fractions were collected manually after passing through the detector. The mild alkaline and temperature conditions enabled successful dissolution of the pectin without inducing any depolymerization or cleavage of glycosidic linkages, although the effect on the methylation and esterification cannot be excluded. This was out of the scope of this study.

### 2.4. Size Distributions and Absolute Molar Mass Determination with SEC-DRI-MALLS

Absolute molar mass determinations were performed by size exclusion chromatography (SEC, SECcurity 1260, PSS, Mainz, Germany) coupled to a multiple angle laser light scattering detector (MALLS, BIC-MwA7000, Brookhaven Instrument Corp., New York, NY, USA) and a refractive index detector (SECcurity 1260, PSS, Mainz, Germany) thermostated at 40 °C. Prior to analyses, 1 mg of the parent pectin sample and the fractions were dissolved in 200 μL of 10 mM NaOH (MilliQ) overnight at room temperature under agitation. Sample injections of 100 μL were performed into a combined column set-up with a SUPREMA pre-column, a SUPREMA 1000 Å, and two SUPREMA 3000 Å analytical columns (PSS, Mainz, Germany) eluting with 1 mL min^−1^ of 10 mM NaOH (MilliQ) as mobile phase at 40 °C. 

Size exclusion chromatography separates macromolecules based on their hydrodynamic volume *V_h_*, which according to the universal calibration theory is directly proportional to the product of the intrinsic viscosity and the number-average molar mass [30,31]. Based on this separation principle, it was possible to perform a universal calibration of our SEC set-up using pullulan standards with known molar masses and their corresponding Mark-Houwink parameters (Equation (1)), and therefore transform the elution volume, which is dependent on the experimental conditions, into hydrodynamic volumes (*V_h_*) or in this case, hydrodynamic radius (*R_h_*), with $V_{h}=\frac{4}{3}\cdot \pi \cdot R_{h}^{3}$. Here, *V_h_* is the hydrodynamic volume; *K* and *a* are the Mark-Houwink parameters; *N_A_* is Avogadro’s number; and *M_n_* is the number-average molar mass.

(1) $$V_{h}=\frac{2}{5}\cdot \frac{K\cdot M_{n}{}^{1+a}}{N_{A}}$$

The pullulan standards (PSS, Mainz, Germany) had molar masses ranging from 342 to 708 × 10^3^ Da (PSS, Mainz, Germany) and the Mark-Houwink parameters for pullulan in aqueous solutions at 40 °C were estimated as *K* = 1.0176 × 10^−3^ dL g^−1^ and *a* = 0.525 [32], whereas the d*n*d*c* was 0.149 mL g^−1^ [33].

After universal calibration of the SEC set-up, the SEC weight distribution *w*(log *V_h_*) for the pectin fractions was obtained from the DRI signal (Equation (2)) [34], whereas the size dependence of the weight-average molar mass *M_w_*(*V_h_*) was obtained from the MALLS data using the Zimm extrapolation (Equation (3)) [35].

(2) $$w(logV_{h})=-S_{DRI}(V_{el})\cdot \frac{dV_{el}(V_{h})}{dlogV_{h}}$$

(3) $$\frac{K_{\theta }\cdot c(V_{h})}{R_{\theta }(V_{h})}=\frac{1}{M_{w}(V_{h})}+\frac{16\cdot \pi^{2}}{3\cdot \lambda }\cdot \frac{1}{M_{w}(V_{h})}\cdot R_{g}^{2}\cdot sin^{2}\left(\frac{\theta }{2}\right)$$

Here *S_DRI_*(*V_el_*) is the DRI signal at each slice of elution volume; *V_el_*(*V_h_*) is the universal calibration curve obtained using pullulan standards and Equation (1); *K_θ_* is a physical constant dependent on the solvent and the scattering conditions; *c*(*V_h_*) is the concentration at each slice of hydrodynamic volume (calculated from DRI), *R_θ_*(*V_h_*) is the excess Rayleigh ratio of scattered light at a certain angle *θ*; *λ* is the wavelength of the incident light in the separation medium; and *R_g_* is the radius of gyration of the macromolecule in solution. Data collection and analysis from SEC separations with light scattering detection was performed using WinGPC software (PSS, Mainz, Germany). A d*n*d*c* of 0.146 mL g^−1^ for pectins in aqueous solutions was employed for the molar mass determinations [36]. 

### 2.5. Carbohydrate Analyses

A combination of methanolysis and TFA hydrolysis according to De Ruiter et al. [37] was used to determine the neutral sugar and uronic acid composition of the samples. Briefly, 2–5 mg of dried sample was heated for 16 h at 80 °C with 1 mL of 2 M HCl in methanol in duplicate experiments. The methanolic HCl was evaporated using air at 25 °C and the remaining carbohydrates were further hydrolyzed with 1 mL of 2 M TFA for 1 h at 121 °C. TFA was removed by evaporation and the samples were dissolved in water in order to be analyzed with HPAEC-PAD. The HPAEC-PAD Dionex ICS-3000 was equipped with a precolumn CarboPac^®^ PA1 4 × 50 mm and a CarboPac^®^ PA1 4 × 250 mm^2^ column. Separation of the neutral and acidic monosaccharides was performed in separate gradients as previously reported [38].

### 2.6. Glycosidic Linkage Analyses

Polysaccharides (5 mg) were first carboxyl reduced with sodium borodeuteride (NaBD_4_) using the method of Kim and Carpita [39]. Carboxyl-reduced polysaccharides in the different fractions (1 mg, four technical replicates) were methylated using the NaOH/CH_3_I method [40]. The methylation step was repeated five times on each sample, thereby avoiding any risk for incomplete methylation. After methylation, the samples were hydrolyzed with 2M TFA at 121 °C for 2 h, reduced, and acetylated [41]. The permethylated alditol acetates were separated and analyzed by GC/EI-MS on a SP-2380 capillary column (30 m × 0.25 mm ID; Sigma-Aldrich, St. Louis, MO, USA) with a temperature program increasing from 160 °C to 210 °C at a rate of 1 °C min^−1^. The mass spectra of the fragments obtained from the permethylated alditol acetates (EI-MS) were compared with those of reference polysaccharides derivatives and to available data [42].

## 3. Results

### 3.1. Fractionation of Bark Pectins and Size Distributions of the Fractions

**Figure 1:**
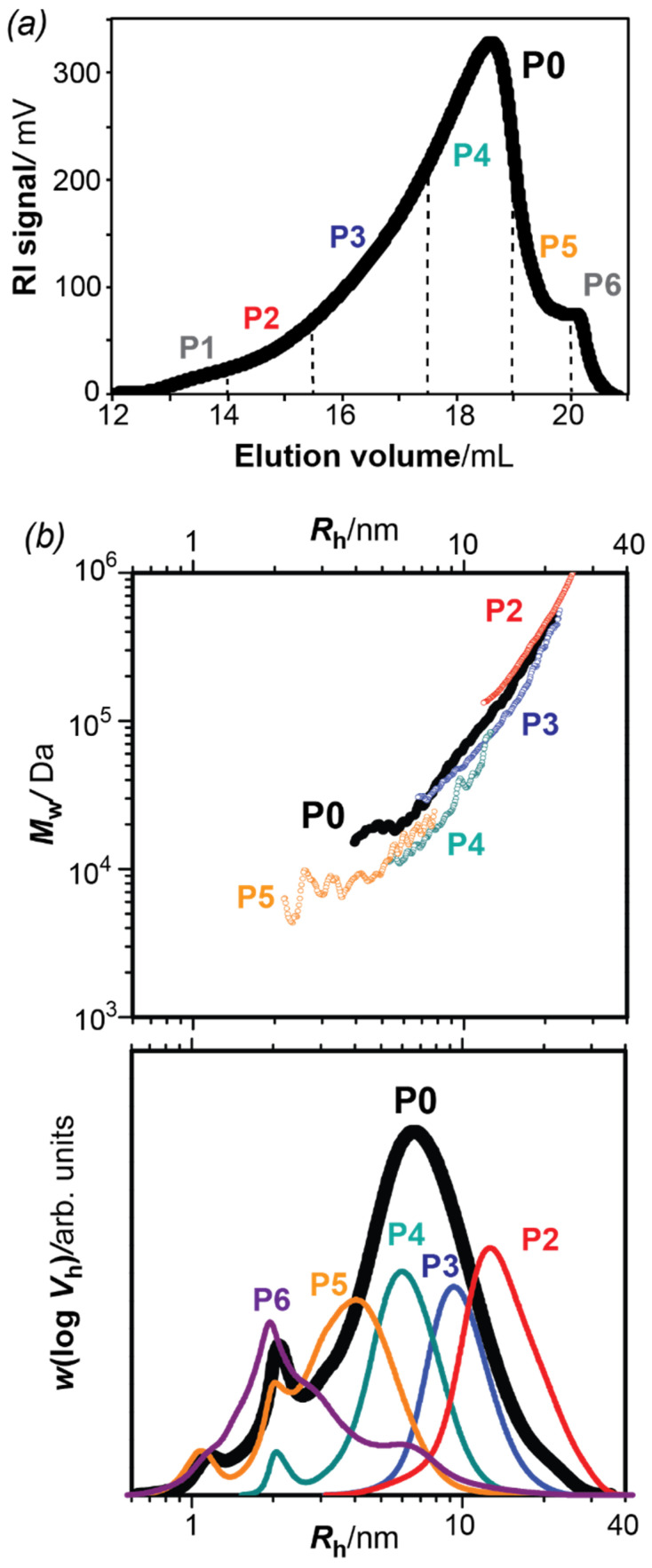
Size fractionation and macromolecules distributions of the spruce bark pectins. (**a**) Preparative size-exclusion chromatography of the parent (P0) pectin sample, resulting in six pectin fractions (P1–P6) with decreasing macromolecular size. (**b**) SEC weight distribution *w*(log *V_h_*) and the size dependence of the weight-average molar mass *M_w_*(*V_h_*) as a function of the hydrodynamic radius (*R_h_*). The *w*(log *V_h_*) distribution and *M_w_*(*V_h_*) data of the fractions P1 and P6 could not be obtained due to the small concentration and molar mass values of these fractions, respectively.

The amylase-treated bark extract (sample P0) was fractionated using preparative size exclusion chromatography (SEC) into six fractions (P1–P6) with decreasing macromolecular sizes (Figure 1a). The yields of the different fractions (Table 1) indicate the larger content in the intermediate fractions (P3-P5) as reflected in the preparative SEC distribution (Figure 1a). The size distributions for each fraction, namely the SEC weight distribution *w*(log *V_h_*) and the size dependence of the weight-average molar mass *M_w_*(*V_h_*), were further determined using analytical size-exclusion chromatography coupled to refractive index detection (DRI) and multi-angle laser light scattering (SEC-MALLS).

Figure 1b represents the SEC weight distributions *w*(log *V_h_*) and the weight-average molar mass *M_w_*(*V_h_*) plotted as a function of the hydrodynamic radius (*R_h_*) for the parent pectin sample (P0) and the resulting fractions. As it can be observed, the parent P0 pectin sample displays a broad distribution with hydrodynamic sizes ranging between 1 and 40 nm. The size fractions (P1–P6) all fell under the envelope of the parent fraction P0 with progressively decreasing hydrodynamic sizes, which evidences the successful preparative fractionation. The SEC weight distributions *w*(log *V_h_*) of the parent pectin P0 and the derived fractions displayed one major peak with symmetrical shape. The shoulders at low *R_h_* (*R_h_* ~ 1 and 2 nm) occurring in all distribution profiles were also present in the eluent blank and were therefore assigned to a system peak and did not affect the molar mass calculations.

Similarly, the absolute weight-average molar mass distributions (*M_w_*) of the different fractions shifted progressively towards lower values, almost superimposing with the parent pectin sample P0. It is worth mentioning that the *M_w_* values of the fractions P1 and P6 could not be obtained due to the small concentration in such fractions. Interestingly, the slope of the size dependence of the molar masses decreased for the smaller fractions. This slope is related to the hydrodynamic compactness of the macromolecules in solution, related to the degree of branching. These results might indicate that the larger macromolecules in fractions P2–P3 had a more compact and dense architecture—typical of branched polymers—compared to the smaller macromolecules in fractions P4 and P5. The intramolecular structure for selected fractions will be verified by linkage analysis and subsequently related to the macromolecular architecture and hydrodynamic properties from SEC-MALLS.

The number- and weight-average molar mass (*M_n_*, *M_w_*), dispersity (*D* = *M_w_/M_n_*) and degree of polymerization (DP) of the fractions are summarized in Table 1. The average molar masses of the original pectin fraction P0 were close to the values previously reported (*M_n_* = 14,600 and *M_w_* = 54,900 Da) for the crude pressurized hot water extract of the inner bark [4]. The molar mass (*M_w_*) and dispersity (*D*) were, however, slightly lower in the present work. This difference might be explained by the amylase treatment performed here, which led to the removal of large starch polymers. The average molar mass of the extracted bark pectins remained low compared to pectins from fruit tissues, which display *M_w_* values in the range of 100,000 to 200,000 Da [43,44,45] or sugar beet pectins (*M_w_* of 500,000 Da) [46]. However, similar *M_w_* were reported for pectins extracted with hot water from different plant sources [47,48,49]. The parent pectin P0 showed a broad dispersity (*D* = 2.2) in comparison to the derived fractions, which displayed a much narrower profile with a dispersity close to unity. Such low dispersity indicated a good fractionation and revealed the obtention of almost monodisperse pectin fractions. The estimated degree of polymerization (DP) of the polysaccharides varied significantly between the fractions, with fraction P3 containing polysaccharide networks four to seven times larger than P4 or P5, respectively.

### 3.2. Carbohydrate Composition

**Figure 2:**
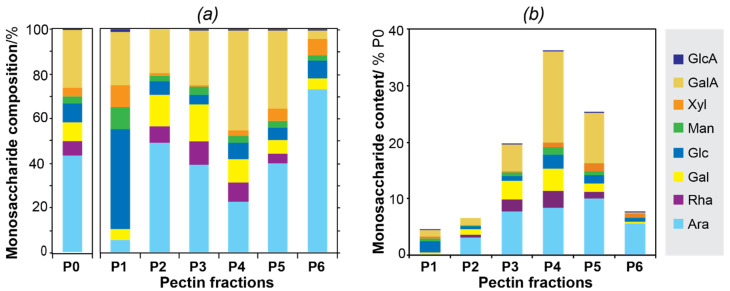
(**a**) Monosaccharide composition (in weight %) of the parent and fractionated pectin samples; (**b**) monosaccharide content of the pectin fractions related to the parent pectin fraction (%P0). NOTE: GlcA (glucuronic acid), GalA (galacturonic acid), Xyl (xylose), Man (mannose), Glc (glucose), Gal (galactose), Rha (rhamnose), Ara (arabinose).

The carbohydrate composition of the parent pectin and the derived fractions (Figure 2a) contained the typical pectic components, including Ara, GalA, Rha, and Gal, albeit with different relative proportions across fractions. Minor presence of other carbohydrates, such as xylose, mannose, and glucose were also detected, which could either be included in the pectin molecules as side chain or be present as individual polysaccharides in minor quantities [13,50]. Interestingly, the first fraction P1 displayed a large abundance of Glc, which could be attributed to residual starch that might have remained after the destarching process. The monosaccharide composition of the remaining pectic fractions (P2–P6) displayed interesting trends related to the relative amounts of GalA and Ara. The fractions with larger macromolecular sizes (P2–P3) showed a high proportion of Ara compared to GalA, accompanied by a higher relative content of Rha and Gal. This suggests the presence of pectic domains rich in arabinans and arabinogalactans linked to Rha residues in rhamnogalacturonan type I (RG-I). The presence of highly branched RG-I domains in the bark of Norway spruce was already proposed [3,4,5,29]. The intermediate fraction (P4) was enriched in GalA (45%), probably originating from linear homogalacturonan domains (HG), with a lower proportion of Ara and Gal moieties. The fraction P5 contained arabinose and galacturonic acid in rather equal amounts (35–40%), with a smaller proportion of Rha. As Rha is considered the branching point that covalently binds arabinans and arabinogalactan side chains with the RG-I backbone, we suggest that P5 is composed of a mixture of individual polysaccharide populations. The fraction P6 had a large proportion of Ara with negligible abundance of GalA, which probably corresponds with free arabinan populations with small hydrodynamic sizes and highly branched topologies. The relative abundance of the monosaccharide content in the different fractions related to the parent P0 pectin fraction is calculated by multiplying the relative amount of the monosaccharide (weight%) found in the fractions with the yield (%) in which the fractions were obtained (Figure 2b). This provides a good overview of the relative abundance of the different pectic domains (e.g., RG-I, HG, arabinan, arabinogalactan) present in the original pectin sample and the derived fractions.

### 3.3. Glycosidic Linkage Analysis

**Figure 3:**
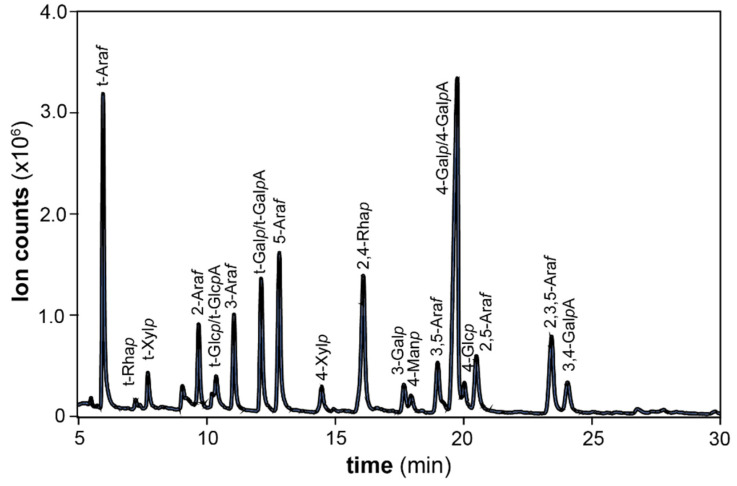
GC-MS chromatogram of the partially methylated alditol acetates (PMAAs) from the pectic fractions (here, P3 as an example) after carboxyl reduction of the uronic acids and subsequent methylation, hydrolysis, reduction and acetylation. The glycosidic linkages were assigned based on the EI-MS spectra and retention time of polysaccharide standards.

The glycosidic linkage composition of the parent P0 and the main SEC fractions (P3–P5) was investigated by methylation and GC-MS analysis (Table 2, Figure 3), in order to study the structural characteristics of the polysaccharides present in spruce bark pectins. Approximately 24–30% of the arabinosyl residues in all fractions occurred as 5-Ara*f* linked units, 31–38% corresponded to terminal arabinosyl residues (t-Ara*f*), and the rest of the arabinose monosaccharides was represented by 3-, 3,5-, 2,5-, and 2,3,5-linked arabinofuranosyl units. These proportions confirmed the occurrence of highly branched arabinans with probably similar structures in P3, P4, and P5. Similar highly branched arabinans were also reported for the pectic polysaccharides from *Picea abies* greenery [51]. A part of the arabinose units, especially the terminal residues, might also arise from the side chains in arabinogalactan associated with galactose units. Two types of arabinogalactans are generally present in pectic polysaccharides, namely arabinogalactans I (AG-I) and II (AG-II). The occurrence of AG-I was confirmed by the presence of 4-linked galactan backbones substituted at position 3. AG-II was also present in the fractions, since 3-, 3,6- and 6-linked galactopyranosyl (Gal*p*) residues were detected.

In all fractions, GalA was mostly composed of 1,4-linked residues (90–93 mol% of total galacturonic acid), which could originate from either RG or HG domains. A similar proportion of 1,4-linked galacturonic acid (ca. 93% of all uronic acid) was reported for acid-extractable polysaccharides from tree greenery of Norway spruce [52]. Approximately 6% of the galacturosyl residues were substituted in position 3 in P3 and P4. This substitution was previously detected in the pressurized hot water extracts of spruce bark [4]. In pectins, galacturonic acid is usually substituted at position 3 with xylosyl residues, forming xylogalacturonan (XGA) chains, or by other sugars such as apiofuranose in the RG-II domain [10]. The presence of RG-I domains was revealed by the signature linkages arising from 2- and 2,4-linked Rha*p* units. These domains were particularly enriched in fractions P3 and P4, as evidenced by the relatively high number of such 2- and 2,4-linked Rha*p* units (~95–98 %mol of the total Rha*p* units). On the contrary, 2-Rha*p* units were absent in the low molar mass fraction P5. Finally, the presence of minor mannan and xylan hemicellulosic populations was revealed by the signature 4-Man*p* and 4-Xyl*p* linkages, respectively. Interestingly, the xylan populations were particularly enriched in the fraction P5.

### 3.4. Pectic Domain Composition. Structural Model for the Pectins in Norway Spruce Bark

**Figure 4:**
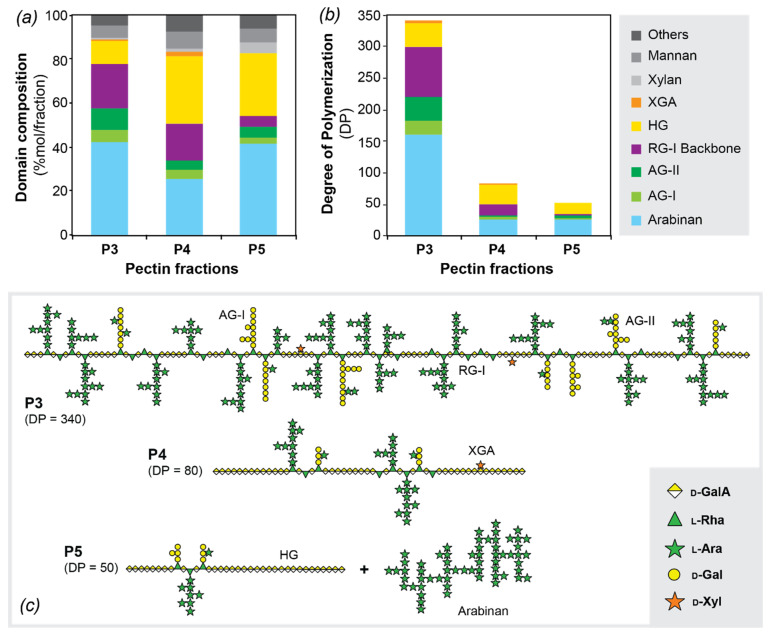
(**a**) Domain composition for each fraction and (**b**) amount of the different pectic domains related to the degree of polymerization obtained by SEC-MALLS. The domains were calculated from the linkage analysis and assigned based on Pettolino et al. (2012) [53]. (**c**) Macromolecular models for the pectin fractions.

The nature of the pectin domains in spruce bark (Figure 4a) was deduced from the glycosidic linkage assignments that are characteristic for the different cell wall polysaccharides [53], as provided in Table 3. The results from the polysaccharide assignment based on the glycosidic linkages were then processed together with the DP obtained with SEC-MALLS in order to estimate the DP of each pectic domain (Figure 4b). This last estimation was based on the hypotheses that all domains were covalently linked to each other and that the pectin distributions in the fractions were monodisperse.

All fractions were composed of approximately 85–90% pectins, where the additional 10–15% were assigned to mannans and xylans (Table 3, Figure 4a). The most significant difference between P3 and the other two fractions (P4–P5) was their differing RG-I and HG content. In fact, the RG-I domain (including side chains) seemed to represent almost 80% of P3, while P4 and P5 contained only 50% of RG-I. On the contrary, HG was present in P4 and P5 in much higher proportions (~30%) than in P3 (~10%). However, the comparison of the average DP of each domain, illustrated in Figure 4b, suggested that the size of the HG domain was similar in P3 and P4. The proportion of HG and RG-I domains strongly depends on the source of the pectin. Commercial citrus and apple pectins, for example, contain mainly HG domains (ca. 65%) and minor amount of RG-I domains (ca. 20–35%) [54,55], whereas in pectins from soybean (*Glycine max*) green tea (*Camellia sinensis*), and potato the RG-I region is the predominant domain [56,57,58]. In the pectic polysaccharide extracted from the greenery of siberian fir (*Abies sibirica*) the main domain detected was also the RG-I domain [59].

The significant change in DP observed between P3 and P4 clearly designated two different pectin populations. On one hand, P3 was composed of high-molar mass branched polysaccharides with a high proportion of branched RG-I domains, whereas P4 had larger abundance of linear polysaccharides with long HG domains. Both fractions occupied, however, relatively similar hydrodynamic volumes since the elution times in SEC were close. Fraction P5 appeared to mainly be composed of arabinan domains, with comparable sizes as in P4, and smaller HG domains. However, the content in RG in this fraction was low, which indicated that some arabinans could be present as individual polysaccharide populations. It has been suggested that pure arabinans could be present in the stem of *Picea abies* [60,61]. Highly branched arabinans with a DP ranging from 34 to 100 were also detected in the pressurized hot water extracts of the bark of several species, such as *Rosa glauca* [62], *Salix alba* L. [63] and *Populus tremuloides* [64].

Here, we propose and illustrate a macromolecular model for the molecular structure for bark pectins, based on the integration of the information on the macromolecular architecture and molecular weight distributions from SEC-MALLS, together with the analysis of the composition and glycosidic linkages of the different pectic domains (Figure 4c). Fraction P3 is mainly composed by heavily branched macromolecules with short HG and RG-I domains, which are distributed block wise along the pectin chains. This representation is based on preliminary data of the degradation pattern of HG with *endo*-polygalacturonase (data not shown). The formation of two narrow peaks at around 5500 kDa and 2500 kDa after incubation with endo-polygalacturonase suggested that the RG domains in the pectin chain had a size of around 15 to 30 sugar units. This proves that the structure of RG-I in P3 is heavily branched by arabinan and arabinogalactan side chains in most of the rhamnose backbone units, as it was reported by methylation linkage analysis. The fraction P4 seems to be formed by less branched macromolecules, where longer unbranched HG domains separate the RG-I regions in the block copolymer. Finally, fraction P5 seems to be composed by individual domains of HG and arabinans that may be linked to the RG-I domains with less probability than in P3 and P4. The origin of the polysaccharides in P5, whether they are present in the native bark or arise as degradation products from the extraction process, remains to be determined. The structural parameters of the pectic polysaccharides, this is, molar mass and branched structure, affect their functional performance in terms of viscosity and other rheological properties. Indeed, higher molar mass directly correlates with an increase of viscosity of pectin solutions and gels [65]. On the other hand, the specific distribution of pectic components (e.g., HG, RG-I, arabinan, and AG) has a deep influence on the rheological properties, also depending on the environment (e.g., pH, ionic strength, presence of low molecular weight cosolutes) [66,67]. Thus, the higher *M_w_* of P3 would suggest higher viscosity compared to P4 and P5. However, the *M_w_* of P3 is still lower than that of other pectins such as citrus pectin. Hence, lower gelation ability could be assumed. Also, the high number of RG-I regions and higher branching in P3 will affect the rheological behavior and result in different properties of P3 compared to P4 and P5. In the past the impact of the HG regions on the rheological properties has mainly been studied. However, there has been increased interest in the use of RG-I-rich pectins, because of their health benefits, and it was shown that RG-I-rich pectins can also form gels with high gel strength [68,69]. The systematic study of the structure—property relations of bark pectins—therefore constitutes the subject of further investigations.

## 4. Conclusions

Here, we provide a structural model for the macromolecular architecture of the pectin populations extracted from the bark of *Picea abies* using pressurized hot water extraction. The size fractionation of the extracted pectins by preparative size exclusion chromatography, followed by monosaccharide and glycosidic linkage analysis, revealed the structural characteristics of the backbone and side chains of the different pectic domains. Different pectic fractions were fractionated from spruce bark, with molecular weights ranging between 5000 and 150,000 Da. The pectins consist of a complex mixture of polysaccharide domains with distinct macromolecular topologies (e.g., hyperbranched and linear domains). The high-molar mass pectic fractions contained mainly hyperbranched RG-I domains with a large abundance of arabinan and arabinogalactan (both type-I and type-II) side chains. The pectic fractions with lower macromolecular sizes were progressively enriched in more linear HG fragments. Finally, highly branched arabinans with DP of 50 were identified as free domains in the pectin fractions with lower molar mass. The obtained structural models will inform the development of target applications for the spruce bark pectins towards the realization of future integral second-generation biorefineries from lignocellulosic feedstocks.

## Figures and Tables

**Table 1:** Average molar mass (*M_n_*, *M_w_*), dispersity (*D*), and degree of polymerization (DP) of the pectic fractions. The yield (% *w*/*w* of P0) for each fraction is also presented.

Fraction	*M_n_* (Da)	*M_w_* (Da)	*D*	*M_ws_*^(a)^ (Da)	DP ^(b)^	Yield (%)
P0	17600	39300	2.2	168	230	100
P1	n.d	n.d	n.d	n.d	n.d	4.5
P2	156,500	240,200	1.5	165	948	6.6
P3	46,100	58,600	1.3	171	340	19.8
P4	13,200	14,500	1.1	175	80	36.2
P5	7000	8700	1.2	163	50	25.2
P6	5600	14,100	2.5	157	36	7.7

^(a)^ Average molar mass of one anhydro sugar based on the carbohydrate composition of the fraction. ^(b)^ Degree of Polymerization (DP) calculated as *M_w_*/*M_ws_*.

**Table 2:** Glycosidic linkage composition (%mol) of the parent pectin (P0) and selected pectin fractions.

Partially Methylated Alditol Acetate (PMAA)	Linkage Type	Short Name	P0	P3	P4	P5
2,3,5-Me_3_-Ara*f*	Ara*f*-(1→	t-Ara*f*	12.6	13.7	10.4	15.0
2,3,4-Me_3_-Ara*p*	Ara*p*-(1→	t-Ara*p*	0.3	0.2	0.3	0.9
3,5-Me_2_-Ara*f*	→ 2)-Ara*f*-(1→	2-Ara*f*	0.5	0.8	0.4	1.8
2,5-Me_2_-Ara*f*	→ 3)-Ara*f*-(1→	3-Ara*f*	4.5	6.8	3.1	5.2
2,3-Me_2_-Ara*f*	→ 5)-Ara*f*-(1→	5-Ara*f*	12.4	12.6	6.5	12.5
2-Me-Ara*f*	→ 3,5)-Ara*f*-(1→	3,5-Ara*f*	3.6	5.3	2.2	3.5
3-Me-Ara*f*	→ 2,5)-Ara*f*-(1→	2,5-Ara*f*	1.7	2.5	1.0	1.7
Acetylated Ara*f*	→ 2,3,5)-Ara*f*-(1→	2,3,5-Ara*f*	5.4	2.3	3.2	4.5
Total Ara			(41.0)	(44.2)	(27.1)	(45.1)
2,3,4,6-Me_4_-Gal*p*	Gal*p*-(1→	t-Gal*p*	3.6	2.8	5.1	1.5
2,4,6-Me_3_-Gal*p*	→ 3)-Gal*p*-(1→	3-Gal*p*	1.2	2.1	1.5	0.2
2,3,6- Me_3_-Gal*p*	→ 4)-Gal*p*-(1→	4-Gal*p*	1.6	3.5	1.5	1.2
2,3,4-Me_3_-Gal*p*	→ 6)-Gal*p*-(1→	6-Gal*p*	2.9	4.9	0.6	1.0
2,3-Me_2_-Gal*p*	→ 4,6)-Gal*p*-(1→	4,6-Gal*p*	0.7	0.9	0.7	0.6
2,6-Me_2_-Gal*p*	→ 3,4)-Gal*p*-(1→	3,4-Gal*p*	0.2	0.3	0.5	0.1
2,4-Me_2_-Gal*p*	→ 3,6)-Gal*p*-(1→	3,6-Gal*p*	1.2	0.9	0.7	1.2
Total Gal			(11.4)	(15.4)	(10.6)	(5.8)
2,3,4,6-Me_4_-Gal*p*A	Gal*p*A-(1→	t-Gal*p*A	2.5	0.4	1.5	2.1
2,3,6- Me_3_-Gal*p*A	→ 4)-Gal*p*A-(1→	4-Gal*p*A	21.4	19.1	36.4	28.4
2,6-Me_2_-Gal*p*A	→ 3,4)-Gal*p*A-(1→	3,4-Gal*p*A	1.7	1.4	2.5	0.0
Total GalA			(25.6)	(20.9)	(40.4)	(30.5)
2,3,4,6-Me_4_-Rha*p*	Rha*p*-(1→	t-Rha*p*	0.6	0.2	0.4	1.8
3,4,6-Me_3_-Rha*p*	→ 2)-Rha*p*-(1→	2-Rha*p*	2.2	3.4	3.5	0.0
3,6-Me_2_-Rha*p*	→ 2,4)-Rha*p*-(1→	2,4-Rha*p*	4.5	6.9	4.7	2.4
Total Rha			(6.3)	(10.5)	(8.6)	(4.2)
2,3,4,6-Me_4_-Glc*p*	Glc*p*-(1→	t-Glc*p*	2.0	0.2	0.9	2.6
2,3,6-Me_3_-Glc*p*	→ 4)-Glc*p*-(1→	4-Glc*p*	2.9	2.4	3.8	2.6
2,3-Me_2_-Glc*p*	→ 4,6)-Glc*p*-(1→	4,6-Glc*p*	2.7	1.4	2.3	0.2
Total Glc			(7.6)	(4.0)	(7.0)	(5.4)
2,3,4-Me_3_-Xyl*p*	Xyl*p*-(1→	t-Xyl*p*	1.4	0.6	1.2	0.8
2,3-Me_2_-Xyl*p*	→ 4)-Xyl*p*-(1→	4-Xyl*p*	2.0	0.6	1.3	5.4
Total Xyl			(3.4)	(1.2)	(2.5)	(6.2)
2,3,6-Me_3_-Man*p*	→ 4)-Man*p*-(1→	4-Man*p*	4.0	3.2	3.3	2.3
2,3-Me_2_-Man*p*	→ 4,6)-Man*p*-(1→	4,6-Man*p*	0.7	0.0	0.0	0.3
Total Man			(4.7)	(3.2)	(3.3)	(2.6)
2,3,4,6-Me_4_-Glc*p*A	GlcA*p*-(1→	t-Glc*p*A	0.3	0.4	0.4	n.d.
2,3,6-Me_3_-Glc*p*A	→ 4)-Glc*p*A-(1→	4-Glc*p*A	0.1	0.2	0.1	n.d.
Total GlcA			(0.4)	(0.5)	(0.5)	(0.2)

**Table 3:** Polysaccharide composition of the bark fractions (calculated based on Pettolino et al. (2012) [53]).

Polysaccharides	Linkage	P3	P4	P5
Arabinan	t-Ara*f*	12.4	9.6	14.2
	3-Ara*f*	6.8	3.1	5.2
	5-Ara*f*	12.6	6.5	12.5
	3,5-Ara*f*	5.3	2.2	3.5
	2,5-Ara*f*	2.5	1.0	1.7
	2,3,5-Ara*f*	2.3	3.2	4.5
	Total	41.9	25.6	41.6
Type I AG	4-Gal*p*	3.5	1.5	1.2
	4,6-Gal*p*	0.9	0.7	0.6
	3,4-Gal*p*	0.3	0.5	0.1
	t-Ara*f*	1.2	0.8	0.5
	t-Gal*p*	0	0.8	0
	Total	5.9	4.3	2.4
Type II AG	3-Gal*p*	2.1	1.5	0.2
	6-Gal*p*	4.9	0.6	1.0
	2-Ara*f*	0.8	0.4	1.8
	3,6-Gal*p*	0.9	0.7	1.2
	t-Rha*p*	0.2	0.4	1.2
	t-Ara*f*/Ara*p*	0.1	0	0
	t-Gal*p*	0.6	0.3	0
	Total	9.6	3.9	5.4
RG I	2-Rha*p*	3.4	3.5	0
	2,4-Rha*p*	6.9	4.7	2.4
	3,4-Gal*p*A	0.8	1.3	0
	4-Gal*p*A	9.5	6.9	2.4
	Total	20.6	16.4	4.8
HG	4-Gal*p*A	9.6	29.5	26
	t-Gal*p*A	0.4	1.5	2.1
	Total	10.0	31.0	28.1
XGA	3,4-Gal*p*A	0.6	1.2	0
	t-Xyl*p*	0.6	1.2	0
	Total	1.2	2.4	0
HX	4-Xyl*p*	0.6	1.3	5.4
	Total	0.6	1.3	5.4
HM	4-Man*p*	3.2	3.3	2.3
	4,6-Man*p*	0	0	0.3
	4-Glc*p*	2.4	3.8	2.6
	4,6-Glc*p*	0	0	0.3
	t-Gal*p*	0	0	0.6
	Total	5.6	7.1	5.8
Others		4.6	8.0	6.5
